# On the Modulatory Roles of Neuregulins/ErbB Signaling on Synaptic Plasticity

**DOI:** 10.3390/ijms21010275

**Published:** 2019-12-31

**Authors:** Ada Ledonne, Nicola B. Mercuri

**Affiliations:** 1 Department of Experimental Neuroscience, Santa Lucia Foundation, Via del Fosso di Fiorano, no 64, 00143 Rome, Italy; mercurin@med.uniroma2.it; 2 Department of Systems Medicine, University of Rome “Tor Vergata”, Via Montpellier no 1, 00133 Rome, Italy

**Keywords:** neuregulins, ErbB receptors, synaptic plasticity, LTP, LTD, hippocampus, midbrain dopamine neurons, dopamine

## Abstract

Neuregulins (NRGs) are a family of epidermal growth factor-related proteins, acting on tyrosine kinase receptors of the ErbB family. NRGs play an essential role in the development of the nervous system, since they orchestrate vital functions such as cell differentiation, axonal growth, myelination, and synapse formation. They are also crucially involved in the functioning of adult brain, by directly modulating neuronal excitability, neurotransmission, and synaptic plasticity. Here, we provide a review of the literature documenting the roles of NRGs/ErbB signaling in the modulation of synaptic plasticity, focusing on evidence reported in the hippocampus and midbrain dopamine (DA) nuclei. The emerging picture shows multifaceted roles of NRGs/ErbB receptors, which critically modulate different forms of synaptic plasticity (LTP, LTD, and depotentiation) affecting glutamatergic, GABAergic, and DAergic synapses, by various mechanisms. Further, we discuss the relevance of NRGs/ErbB-dependent synaptic plasticity in the control of brain processes, like learning and memory and the known involvement of NRGs/ErbB signaling in the modulation of synaptic plasticity in brain’s pathological conditions. Current evidence points to a central role of NRGs/ErbB receptors in controlling glutamatergic LTP/LTD and GABAergic LTD at hippocampal CA3–CA1 synapses, as well as glutamatergic LTD in midbrain DA neurons, thus supporting that NRGs/ErbB signaling is essential for proper brain functions, cognitive processes, and complex behaviors. This suggests that dysregulated NRGs/ErbB-dependent synaptic plasticity might contribute to mechanisms underlying different neurological and psychiatric disorders.

## 1. Introduction

Neuregulins (NRGs) are a family of neurotrophic factors, which are essential for proper development of the peripheral and central nervous system, as well as adult brain homeostasis. NRGs were discovered more than 25 years ago, independently by four groups that identified a protein, now recognized as the first member of the NRGs family, neuregulin 1 (NRG1), as a factor able to activate ErbB2 tyrosine kinase receptors (called heregulin or neu differentiation factor (NDF)) [1,2,3], to induce proliferation of Schwann cells (called glial growth factor (GGF)) [4,5,6,7,8], or to stimulate the synthesis of acetylcholine receptors at developing neuromuscular junctions (NMJ) (called acetylcholine receptor inducing activity (ARIA)) [9]. Such a diverse array of names and corresponding functions of NRG1 are indicative of the versatility and importance of NRGs in human brain. Actually, far from their first identification, now it is recognized that NRGs, by acting on ErbB tyrosine kinases (ErbB2, ErbB3, and ErbB4), are crucial developmental factors, mediating neural differentiation, neuronal guidance, myelination, synapse formation, and NMJ development, as well as they represent important modulators of neuronal excitability, neurotransmission, and synaptic plasticity in adult brain. By these multifaceted roles, NRGs/ErbB signaling controls key physiological neural functions, affecting learning and memory processes and complex behaviors, and its dysfunction is emerging as a pathological feature in different neurological and psychiatric disorders, including schizophrenia, bipolar disorder, autism spectrum disorders, Alzheimer’s disease, major depressive disorder, addiction, Parkinson’s disease, and peripheral and central nervous system injury diseases.

In this review, we will provide an overview of NRGs/ErbB roles in the regulation of synaptic transmission, by focusing on its contribution to the control of synaptic plasticity in the hippocampus and midbrain dopamine (DA) nuclei. Available evidence supports diverse mechanisms engaged by NRGs/ErbB receptors in the regulation of long term modifications of synaptic transmission in these brain areas, depicting a complex scenario involving either presynaptic or postsynaptic NRGs-mediated mechanisms, which affect glutamatergic, GABAergic, and DAergic transmission.

## 2. Neuregulins and ErbB Receptors: Subtypes and Signaling Pathways

### 2.1. Neuregulins

**Figure 1:**
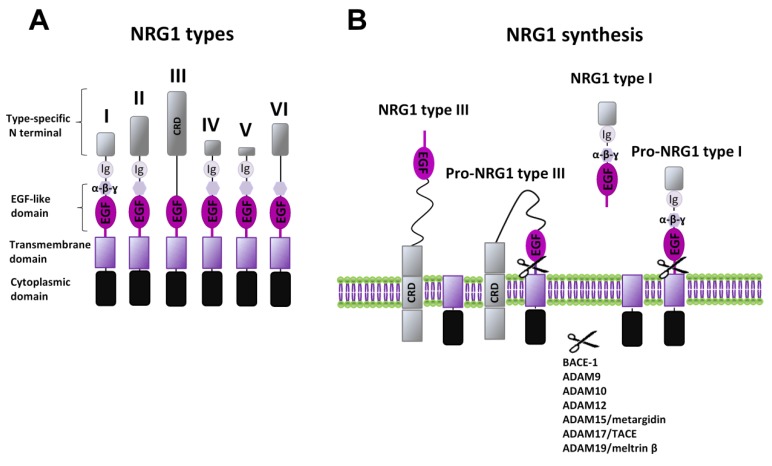
NRG1 types and synthesis. (**A**) Diagram of structural differences of various NRG1 types. (**B**) Synthesis of NRGs’ mature forms is induced by different ADAMs and MMPs, with the release of soluble forms (as for example for NRG1 type I) or the exposure of the membrane-anchored EGF-like domain (as for NRG1 type III).

Neuregulins (NRGs) are a family of epidermal growth factor (EGF)-related proteins encoded by six genes (*Nrg1–Nrg6*). Each gene, by specific controlled transcription and splicing mechanisms, typically produces many mRNA and protein isoforms all expressing a shared domain, the EGF-like domain, required for signaling activation. Neuregulin 1 (NRG1) is the most extensively studied and well characterized member of the NRGs family. The *Nrg1* gene produces six different types (NRG1 I–VI) and 33 spliced isoforms, due to specific uses of six different transcriptional initiation sites and by alternative splicing [10,11,12,13,14]. NRG1 types (I–VI) are identified based on differences in the N terminal domain, besides the presence of immunoglobulin (Ig)-like domains, and/or a cysteine-rich domain (CRD) (Figure 1A). NRG1 type I, II, IV, and V isoforms have the Ig domain, which contributes to distinct interactions with extracellular matrix components (e.g., heparan-sulfate proteoglycans (HSPGs)) and defines the distance and concentration over which these growth factors act [15]. NRG1 type III is the only presenting a CRD, which serves as a secondary transmembrane domain, and thus is a membrane-anchored isoform, acting in an autocrine manner.

NRG1 type I is also known as heregulin, Neu differentiation factor (NDF), or acetylcholine receptor inducing activity (ARIA) [1,2], whereas types II and III have been identified as glial growth factor (GGF) [6], and sensory and motor neuron derived factor (SMDF), respectively [16], based on the first function/cellular population for which they were firstly identified.

NRG1 types are widely and diffusely expressed in both central and peripheral nervous system, as well as in heart, liver, stomach, lung, kidney, spleen, and skin. Human brain areas showing higher NRG1 expression are prefrontal and cingulate cortex, hippocampus, habenula, amygdala, substantia nigra, dorsal and ventral striatum (caudate, putamen, and nucleus accumbens), hypothalamus, spinal cord, and cerebellum [14,17]. All six types of NRG1 are detectable in brain, but the abundance of each form varies significantly, being also related to the developmental period and neuronal activity [14]. In general, NRG1 type III appears to be the predominant brain type of NRG1, while type I and II NRG1 are less expressed, similar to type IV, which, however, is brain specific.

The *Nrg2* gene produces two types of NRG2s, with different EGF-like domains, termed NRG2α and NRG2β, respectively, and at least 10 isoforms due to alternative splicing [18,19]. NRG2 is expressed in the developing nervous system and is also found in the embryonic heart, lung, and bladder [19,20]. In adult brain, NRG2′s highest expression is in hippocampal dentate gyrus (granule cells), cerebellum, and olfactory bulbs [18,19,20], whereas weaker expression has been reported in the neocortex, hippocampal CA1–CA3 neurons, and striatum [21].

NRG3 is present in different splicing forms, up to 15 [22,23,24,25], and is mainly diffused in the brain, either in the embryonic or adult stage. NRG3 has been detected in spinal cord and numerous brain regions, including anterior olfactory nucleus, cerebral cortex, piriform cortex, vestibular nuclei, medial habenula, hypothalamus, thalamus, deep cerebellar nuclei, and hippocampus [26].

NRG4 exists in five isoforms [27,28]. Different from the other NRGs, NRG4 expression appears more confined to peripheral organs, being expressed at high levels in the pancreas, in the skeletal muscle, and in the brown adipose tissue, with its expression in adult brain considered negligible [27,29]. Recently, however, NRG4 expression has been reported in the developing brain, in various brain areas like cortex, hippocampus, cerebellum, olfactory bulb, midbrain, and brain stem [30].

NRG5 is also known as tomoregulin or transmembrane protein with EGF-like and two follistatin-like domains 1 (TMEFF1) [31,32]. NRG5 has five spliced isoforms [27,28], and it is highly expressed in several brain areas, olfactory bulb, amygdala, different regions of cortex (entorhinal, cingulate, motor, somatosensory cortex), various areas of hippocampus (CA3, CA1, and subiculum), locus coeruleus, substantia nigra pars compacta (SNpc), hypothalamic nuclei (ventromedial and paraventricular), and cerebellum [33].

NRG6 is also called neuroglycan C (NGC), or chondroitin sulfate proteoglycan 5 (CSPG5), or chicken acidic leucine rich EGF-like domain containing brain protein (CALEB) [32,34]. In adult human brain, the strongest NRG6 expression has been reported in the striatum (caudate and putamen nuclei), hippocampus, amygdala, and cerebral cortex, whereas weaker expression has been reported in substantia nigra, thalamus, pons, medulla oblongata, and cerebellum [35].

### 2.2. NRGs Processing

NRGs are synthesized as precursors, called pro-NRGs, being the active forms produced following a proteolytic shedding. Different proteases have been associated with the NRGs’ proteolysis, mainly belonging to the “A disintegrin and metalloprotease” (ADAM) subfamily of the matrix metalloproteinases (MMPs), like ADAM9, ADAM10, ADAM12, ADAM15 (also called metargidin), ADAM19 (also called meltrin-β), and ADAM17 (also called tumor necrosis factor-α converting enzyme (TACE-1)). In addition, NRGs are processed by the β-amyloid converting enzyme (BACE-1), a ɣ-secretase that is better known for its involvement in the synthesis of β-amyloid peptides. Different NRGs can have distinct mechanisms regulating their maturation and release. More detailed information is available for NRG1. Almost all types of NRG1 are secreted as soluble factors, released after the conversion from pro-NRG1 to mature NRG1 forms, thus acting in a paracrine manner (Figure 1B). The diffusion and specificity of action on distinct cellular populations might be also allowed by type specific domains (Ig-like or additional spacer domain, as well as differences in N terminus). NRG1 type III, also after its conversion to the mature form, remains attached to the cell membrane due to its CRD, and thus, it acts in an autocrine manner, signaling only to immediately neighboring cells [13,36] (Figure 1B). Regarding peculiar mechanisms that might control the synthesis of specific NRGs, it has been reported that NRG1 types I and II or NRG2 accumulate as pro-forms on cell bodies and are released by MMPs, in an activity dependent manner (e.g., following NMDAR activation), whereas NRG1 type III and NRG3 seem to be constitutively processed by BACE and accumulate on axons where they interact with ErbB in juxtacrine mode [37].

Production of mature NRGs, by ectodomain shedding, occurs in response to diverse stimuli, and possibly in a type specific and area related way. NRG1 levels are increased by an enhancement of neuronal activity, as the one prompted during epileptic seizures in a rat model of epilepsy induced by kainic acid administration, as well as by milder neuronal activities, as induced following forced locomotor activity [38]. Interestingly, neuronal activity seems to shape in a different way the expression of distinct NRG1 types/isoforms, since epileptic seizures appear to not alter NRG1 type II and III levels, while strongly increasing NRG1 type I expression [14]. Increased expression of NRG1 type II after seizure insurgence appears instead in non-neuronal cells. In this regard, a depolarizing treatment increases NRG1 type I expression also in astrocytes from cellular cultures, thus suggesting that brain activity might differently regulate the levels of distinct NRG1 isoforms in diverse cellular populations/brain regions, thus contributing to compartmentalized NRGs related actions.

Moreover, production of soluble NRGs, by ectodomain shedding, occurs in response to other stimuli, like the activation of G protein coupled receptors (GPCR) or ionotropic receptors, through a process that ultimately leads to ErbB transactivation. Such mechanisms involve the activation of MMPs and/or ADAM-dependent shedding of pro-NRGs as a consequence of GPCRs activation, such as angiotensin II receptors, protease-activated receptor 1 (PAR-1), as well as NMDAR stimulation [37,39]. Interestingly, the interplay between NRGs and proteases is bidirectional. Indeed, it has been seen that NRG1 increases the expression of MMP-9, a metalloproteinase critically involved in the regulation of extracellular matrix regulation [40].

### 2.3. ErbB Receptors

NRGs signal by activating tyrosine kinases receptors of the ErbB family. Four ErbB subtypes (ErbB1–4) have been identified, of which ErbB2, ErbB3, and ErbB4 mediate NRGs signaling, whereas ErbB1, also known as the epidermal growth factor (EGF) receptor, is activated by EGF. Upon NRGs binding, ErbB receptors undergo a structural/conformational change in the juxta-membrane region, which potentiates the affinity for other ErbB subunits, thus promoting the formation of homo- and/or hetero-dimers of functional ErbB receptors.

Each ErbB subunit displays a peculiar profile, regarding ligand binding properties and affinities or catalytic activity, which confer them distinct abilities in the possibility to form homodimers or heterodimers. ErbB4 represents the only autonomous subunit, since it either expresses NRGs binding sites or has active kinase domains, thus possibly forming both homodimers and heterodimers of ErbB receptors. ErbB3 has ligand binding sites, but does not express an active kinase domain [41], thus, it cannot form homodimers, nor directly phosphorylate other ErbB receptors, but can associate with other ErbB subunits (ErbB2 and ErbB4) in the formation of heterodimers. ErbB2, instead, has an active kinase domain, but does not bind NRGs or other identified ligands (being for this still considered an orphan receptor). Despite this, ErbB2 represents the preferred dimerization partner among all ErbB receptors [42], and dimerization with ErbB2 strongly increases NRGs’ binding affinity for ErbB3 and ErbB4 [43,44]. Hence, NRGs actions are mediated by heterodimers ErbB2/ErbB3, ErbB2/ErbB4, and ErbB3/ErbB4, as well as ErbB4/ErbB4 homodimers.

Different NRGs display specific profiles of affinity for different ErbB subunits. NRG1 and NRG2 bind both ErbB3 and ErbB4, thus possibly activating all ErbB dimers (ErbB2/ErbB3, ErbB2/ErbB4, ErbB3/ErbB4, ErbB4/ErbB4), whereas NRG3 and NRG4 only bind to ErbB4; thus, they signal through ErbB4/ErbB4 and/or ErbB2/ErbB4 dimers. Notably, NRG3 has a much higher affinity for ErbB4 than any other NRGs [45]. Despite NRGs are not ligands for ErbB1, it can form heterodimers with ErbB4 [46,47,48], thus implying that NRGs signaling can also be mediated by ErbB1/ErbB4. However, evidence suggests that the activation of ErbB1/ErbB4 causes significantly different cellular effects with respect to ErbB1/ErbB1 homodimers (which mediate specific EGF effects), hence still implying segregated functions of EGF and NRGs in neurons [13,46,49,50,51].

### 2.4. NRG/ErbB Signaling

**Figure 2:**
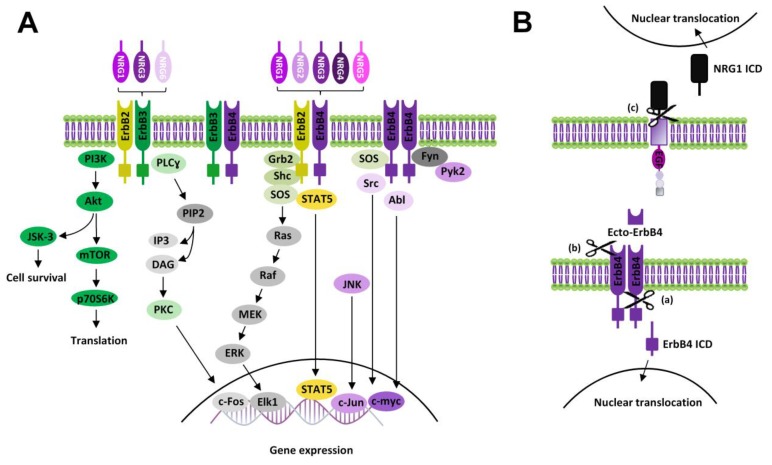
NRGs/ErbB signaling. (**A**) Diagram showing canonical ErbB signaling pathways activated by NRGs. (**B**) Scheme of non-canonical ErbB signaling modalities: (a) Non-canonical forward ErbB4 signaling: proteolytic cleavage of ErbB4 causes the release of its intracellular domain (ErbB4 ICD), which translocates to the nucleus, modulating gene expression; (b) Ecto-ErbB4 domain, containing the binding site of NRGs, is released by proteolytic cleavage of ErbB4 extracellular domain. Ecto-ErbB4 can bind pro-NRGs, thus interfering with NRGs canonical signaling or triggering NRG1 backward signaling; (c) NRG1 backward signaling: proteolytic cleavage of NRG1 (pro-NRG1s or membrane-anchored NRG1 type III) in the intracellular domain induces the release of NRG1 ICD, which triggers backward signaling by nuclear translocation and modulation of gene transcription.

In the canonical ErbB signaling (Figure 2A), NRGs binding induces a conformational change of ErbB subunits fostering trans-phosphorylation and the consequent recruitment of proteins containing phosphotyrosine binding or Src homology-2 (Shc-2) domains, which act as adaptor/effector molecules, triggering the activation of multiple signaling pathways. Intracellular pathways commonly activated by NRGs, downstream of ErbB receptors, are PI3K-Akt-mTOR-S6K and Ras-Raf-MEK-ERK [13]. Other NRGs/ErbB-activated pathways include the PLC-PKC pathway, as well as kinases like c-Abl, JNK, CDK5, Kyn, and Pyk2 [52,53,54]. NRGs-induced effects that involve protein synthesis and cause neuronal growth and survival are mainly mediated by the stimulation of PI3K-Akt-mTOR and glycogen synthase 3 kinase (GS3K), activated downstream of Akt. Phosphatydil-inositole-3 kinase (PI3K) catalyzes the phosphorylation of phosphatidylinositol 4,5-diphosphate (PIP2) to the second messenger phosphatidylinositol 3,4,5-triphosphate (PIP3), which fosters the activation of the protein serine/threonine kinase Akt, then activating the mammalian target of rapamycin (mTOR) (Figure 2A). Among ErbB subunits, ErbB3 expresses the highest prevalence of docking sites for PI3K [55]; thus, the activation of the PI3K-Akt-mTOR pathway might be preferentially due to the stimulation of ErbB3-containing receptors, like ErbB2/ErbB3 and/or ErbB3/ErbB4.

Ras-Raf-MEK-ERK is another pathway commonly stimulated by NRGs/ErbB receptors. NRGs-induced activation of Ras-Raf-MEK-ERK is allowed by the recruitment of the adaptor protein, the growth factor receptor/bound protein 2 (GRB2) to the phosphotyrosine residues of activated ErbB subunits. GRB2 binding with ErbB can be either direct or intermediated by the interaction with the Src homolog and collagen homolog (SHC) adaptor protein, which directly binds tyrosine phosphorylated ErbB sites, thus being itself phosphorylated and then binding GRB2. ErbB-GRB2 then recruits and activates Son of Sevenless (SOS), a guanine nucleotide exchange factor, which fosters GTP availability for binding to Ras, thus activating this kinase. Active Ras starts the kinase cascade pathway including c-Raf, MEK1/2, and ERK1/2. Phosphorylated ERK1/2 translocates to the nucleus, where it activates transcriptional factors (like Elk1), inducing the transcription of genes promoting cell growth and survival. A residual pool of active cytoplasmic ERK1/2 also phosphorylates cytoskeletal proteins such as actin, which promotes cell motility, regulators of cell division and cytokines, vesicle, and organelle movement, and mitochondrial targets such as Bcl2 that render cells resistant to apoptosis (Figure 2A). Other NRGs/ErbB-activated pathways, like the PLC-PKC pathway or c-Abl, JNK, CDK5, Kyn, and Pyk2 kinases [52,53,54], are mainly involved in the regulation of gene expression, by controlling the activity of transcriptional factors such as c-Fos, Elk1, STAT, c-Jun, and c-Myc.

Besides canonical ErbB activation, NRGs/ErbB-dependent effects can be mediated by additional signaling modalities, i.e., “non-canonical forward ErbB signaling” and “NRG1 backward signaling” (Figure 2B). Non-canonical ErbB signaling is started by proteolysis of ErbB4, that can be subjected to cleavage, by ɣ-secretase, in the membrane-bound fragment with the release of the ErbB4 intracellular domain (ErbB4-ICD) [56,57]. Such ErbB4-ICD can translocate to the nucleus, thus regulating gene transcription [58] (Figure 2B). ErbB4 can be also cleaved by TACE in the extracellular membrane region with the release of a soluble peptide (called ecto-Erb4), which contains the NRG1 binding site and acts as a ligand for membrane-anchored NRGs (i.e., immature pro-NRGs forms or the membrane-bound form of NRG1 type III). Ecto-ErbB4 binding to presynaptic NRGs has dual effects, since either it neutralizes NRGs’ canonical actions by preventing NRGs/ErbB forward signaling or can trigger the so-called “NRG1 backward signaling” [59] (Figure 2B). Such a modality of non-canonical NRG1 signaling is elicited by a proteolytic cleavage of the intracellular domain of pro-NRG1, by ɣ-secretase, with the release of the NRG1 intracellular domain (NRG1-ICD). After its translocation to nucleus NRG1-ICD, through the interaction with the transcription factor Eos, regulates the transcription of different genes, including postsynaptic density (PSD) protein PSD95 [13,60]. Some evidence also documented that NRG3, like NRG1, can participate in back signaling by the C-terminal domain [59].

Overall, the emerging scenario demonstrates that NRGs can activate a complex network of signaling pathways, since NRGs/ErbB signals can be transmitted either in forward (canonical and non-canonical) and backward modalities (Figure 2A,B).

## 3. NRGs/ErbB Roles in the Modulation of Synaptic Plasticity

### 3.1. NRGs/ErbB-dependent Regulation of Synaptic Plasticity in the Hippocampus

Synaptic plasticity, the ability to finely adjust the strength of synaptic transmission, is a critical feature of the CNS and is supposed to underlie essential physiological processes, such as learning and memory, as well as complex behaviors, including goal-oriented behaviors. Hippocampal synapses operate over a dynamic range of efficacy and are subject to both short- and long-term forms of plasticity, including long-term potentiation (LTP) and long-term depression (LTD), leading candidates as synaptic mechanisms for memory [61,62,63,64]. While the description of different types of synaptic plasticity in the hippocampus is out of the scope of this review, here we will focus on those that are known to be modulated by NRGs/ErbB signaling.

#### 3.1.1. Glutamatergic LTP at CA3–CA1 Synapses

LTP at CA3–CA1 synapses of the hippocampus is the most extensively studied form of activity-dependent synaptic plasticity in the vertebrate nervous system. LTP induction can be triggered by different stimulation protocols, resembling those occurring physiologically, and it mainly requires NMDARs activation during a strong postsynaptic depolarization. The increase in postsynaptic calcium concentration leads to the consequent activation of biochemical events necessary to sustain LTP expression and maintenance, which is ultimately due to increased AMPAR-mediated excitatory postsynaptic currents (EPSCs) [61].

Stimulating protocols able to induce hippocampal LTP include high-frequency (tetanic) stimulation (HFS, 100 Hz) or “pairing protocols”, during which delivery of low-frequency synaptic activation is paired with a direct depolarization of the postsynaptic cell. LTP induction is also achieved by a stimulation that generates a synaptic response within a discrete time window prior to the firing of the postsynaptic cell, producing the so called “spike-time dependent plasticity” (STDP) [65,66], as well as through the delivery of theta-burst stimulation (TBS), which consists of a pattern of neuronal firing (complex spikes) applied at the frequency of the hippocampal theta rhythm, which spontaneously occurs during behavior [67]. Depotentiation of potentiated synapses, mimicking an LTP reversal, is an additional mechanism preserving synaptic homeostasis at hippocampal CA3–CA1 synapses. Such depotentiation can be induced in acute hippocampal slices and in freely moving animals by brief TBS, delivered shortly after LTP induction [68].

The first demonstration that NRGs/ErbB signaling plays a central role in the modulation of synaptic plasticity in adult brain is represented by the evidence that NRG1 application to hippocampal rat slices prevents the induction of LTP at Schaffer collaterals-CA1 synapses [69]. In particular, a treatment with NRG1 suppresses the induction of HFS-induced LTP of field excitatory postsynaptic potentials (fEPSPs) from the CA1 dendritic region, without affecting basal glutamatergic synaptic transmission [69]. NRG1′s effects on tetanic-induced LTP are concentration dependent and considered to be reliant on postsynaptic mechanisms, since paired-pulse facilitation of evoked glutamatergic synaptic responses, representing a measure of presynaptic function, was not altered by NRG1 treatment [69]. The involvement of NRGs/ErbB signaling in the modulation of hippocampal synaptic plasticity has been further confirmed by several other pieces of evidence, also providing insights into the cellular mechanisms engaged by NRGs in the maintenance of synaptic efficacy and neuronal connectivity in hippocampal formation. NRG1 acutely reverses TBS-induced LTP of fEPSPs in the hippocampal CA1 area at pre-stimulation values [70]. Such NRG1-dependent depotentiation depends on the activation of ErbB receptors, since it is counteracted by ErbB inhibitors. Notably, besides preventing NRG1-dependent synaptic depotentiation, ErbB inhibition per se increases the magnitude of TBS-induced LTP, thus supporting a role of endogenous ErbB signaling in synaptic strength regulation. NRG1-induced synaptic downscaling appears to be mediated by a specific internalization of AMPARs, whereas NMDARs surface expression and function are not altered [70].

**Figure 3:**
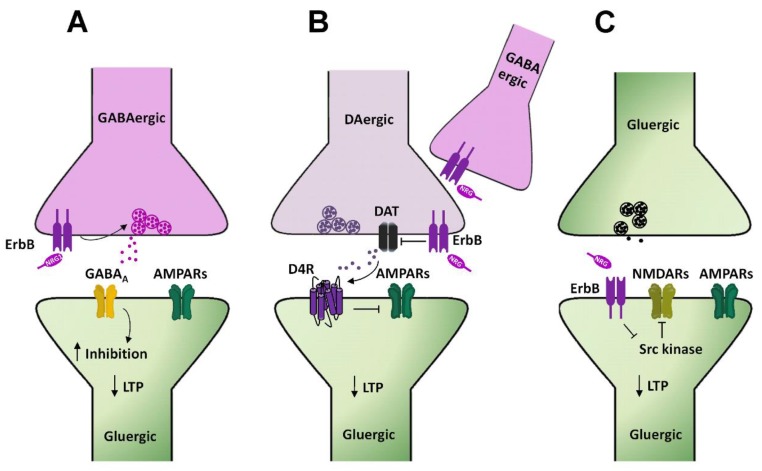
NRGs/ErbB-dependent regulation of LTP in the hippocampal CA1 area. (**A**–**C**) Diagrams illustrating the cellular mechanisms underlying NRGs/ErbB-dependent impairment of LTP at hippocampal CA3–CA1 synapses. (**A**) NRG1-dependent activation of ErbB4 in GABAergic interneurons, by triggering GABA release, fosters the activation of GABA_A_ receptors expressed in CA1 pyramidal cells, thus reducing neuronal excitability and dampening LTP induction. (**B**) NRG1-induced ErbB4 activation on DAergic terminals inhibits DAT activity, thus increasing DA extracellular levels. The consequent activation of D4 receptors localized on CA1 pyramidal neurons impairs LTP induction/expression, via internalization of AMPARs. (**C**) NRG1-dependent ErbB4 activation in CA1 pyramidal neurons inhibits Src kinase and consequently NMDAR activity, thus reducing LTP expression.

Although the precise cellular mechanisms by which NRG1 prevents LTP induction [69] or depresses its expression [70] at hippocampal CA3–CA1 synapses are not completely elucidated, several hypotheses have been postulated, mainly based on evidence from studies analyzing synaptic plasticity at CA3–CA1 synapses following conditional ErbB genetic ablation or pharmacological inhibition. GABAergic transmission plays an important role in the NRG1-dependent suppression of LTP at CA3–CA1 synapses in the hippocampus, as proven by the evidence that NRG1′s effects on LTP are impaired in mice harboring a conditional genetic ablation of ErbB4 in parvalbumin (PV) expressing GABAergic interneurons [71]. NRG1 has a facilitatory effect on GABAergic transmission, since it increases GABA_A_-mediated currents in CA1 pyramidal cells, as a consequence of a direct stimulation of ErbB4 located on PV+ GABAergic interneurons. Thus, by boosting GABA-mediated inhibition in CA1 pyramidal neurons, NRG1 could counteract LTP induction/expression at CA3–CA1 synapses. Interestingly, since the conditional deletion of ErbB4 in CAMKII positive neurons (i.e., pyramidal neurons in the forebrain) does not alter the NRG1-induced suppression of LTP, it has been suggested that ErbB4 in CA1 pyramidal cells has a minor contribution to the regulation of glutamatergic LTP [32,71]. Thus, the proposed scheme accounting for this evidence points to a direct stimulation, by NRG1, of ErbB4 receptors expressed on PV+ GABAergic neurons, which enhances GABA release on CA1 pyramidal cells, thus increasing their inhibition and shifting up the threshold for LTP induction at CA3–CA1 synapses (Figure 3A). Notably, NRG1/ErbB-dependent LTP impairment has been observed also in the presence of GABA_A_ antagonists [70,72], thus questioning NRG1/ErbB-induced GABA release acting on the GABA_A_ receptor as specific mechanism underlying LTP impairment. Thus, additional mechanisms, independent on GABA_A_ stimulation, might be engaged by NRGs/ErbB signaling in LTP depression [32].

Besides GABAergic transmission, also dopamine (DA) transmission takes part in NRG1-dependent modulation of LTP at hippocampal CA3–CA1 synapses. NRG1 injection in the dorsal CA1 area of the hippocampus of rats acutely stimulates DA release, with the consequent activation of DAergic D4 receptors, which directly mediate the inhibition of glutamatergic synaptic plasticity at CA3–CA1 synapses [73]. Indeed, NRG1-dependent reversal of LTP is selectively blocked in hippocampal slices treated with a D4 antagonist, L-745,870 [73]. The functional role of D4 in shaping hippocampal synaptic plasticity at CA3–CA1 synapses, as well as its engagement in NRG1-dependent regulation of LTP have been further confirmed by experiments showing that D4 pharmacological activation mimics NRG1’s effects on LTP, which are conversely abolished in D4 knockout (KO) mice [73]. However, while this evidence suggests involvement of DA transmission in NRGs/ErbB-dependent LTP regulation at hippocampal CA3–CA1 synapses, the specific mechanisms underlying NRG1-induced DA release in the CA1 dorsal hippocampus are not completely clarified yet. In this respect, it has been previously postulated that NRG1-induced ErbB activation in GABAergic PV+ interneurons might be instrumental to NRG1-induced DA release [32,73]. This hypothesis is mostly based on the preferential localization of ErbB4 on GABAergic PV+ interneurons in the hippocampus and the evidence that ErbB4 activation is involved in LTP modulation. Nevertheless, a functional link by which ErbB4 stimulation of GABAergic interneurons could lead to increased DA extracellular levels is lacking. More recently, such a contribution of GABAergic interneurons has been questioned by the evidence that NRG1-dependent regulation of DA outflow in CA1 dorsal hippocampus is dependent on ErbB4 expressed on DA neuronal terminals [74]. Indeed, in mice harboring a conditional genetic ablation of ErbB4 in tyrosine hydroxylase (TH) positive neurons, NRG1 injection in CA1 dorsal hippocampus fails to potentiate DA release [74]. Regarding the underlying functional mechanisms, NRG1-dependent regulation of DA levels appears to be mediated by an interplay between ErbB4 and the dopamine transporter (DAT) expressed on DAergic terminals. Indeed, based on the evidence that ErbB4 stimulation inhibits DAT activity in neuronal cultures, it has been proposed that NRG1-induced potentiation of DA transmission is consequent to an ErbB4-induced DAT inhibition [74]. Hence, according to this hypothesis, hypofunctional DA reuptake results in increased DA extracellular levels, which fosters D4 activation in CA1 pyramidal neurons, depressing LTP at CA3–CA1 synapses (Figure 3B). Regarding the specific involvement of D4 receptors downstream of NRG1-induced DA release, however, there is recent evidence demonstrating that, although either NRG1/ErbB signaling or D4 stimulation is able to mediate LTP reversal/suppression, they act in an independent way in the modulation of glutamatergic synaptic plasticity in the CA1 dorsal hippocampus [75].

NRG1-dependent LTP depression might also be induced by additional postsynaptic mechanisms, directly occurring in hippocampal CA1 pyramidal neurons. It has been reported that NRG1/ErbB4 signaling interferes with the Src-dependent regulation of NMDAR in CA1 pyramidal neurons [72]. Specifically, NMDAR-mediated transmission in CA1 pyramidal neurons is enhanced by activating Src kinases, as demonstrated by the injection of Src activating peptides in single pyramidal neurons [72]. Such Src-dependent potentiation of NMDAR-mediated transmission is impaired by NRG1/ErbB4 signaling, which directly inhibits Src kinase activity. Hence, a model accounting for this evidence depicts ErbB4 activation in CA1 pyramidal cells as the triggering event to reduce, in a cell autonomous manner, NMDAR-mediated transmission and consequently impairing LTP expression (Figure 3C). Notably, ErbB4 localization in the hippocampus is more restricted to GABAergic interneurons [76,77,78], while expression in CA1 pyramidal cells appears low [79,80,81,82] or absent [76,77,78]. This questions the postsynaptic/cell autonomous function of ErbB4 in the NRGs-dependent LTP impairment at CA3–CA1 synapses. Notwithstanding, in spite of undetected expression, ErbB4 functions in hippocampal and cortical pyramidal neurons have been reported [30,72,83,84,85,86]. Thus, the factual role of ErbB4 in hippocampal CA1 pyramidal cells in NRGs-dependent LTP regulation remains to be better elucidated.

#### 3.1.2. mGluRI-dependent Glutamatergic LTD

**Figure 4:**
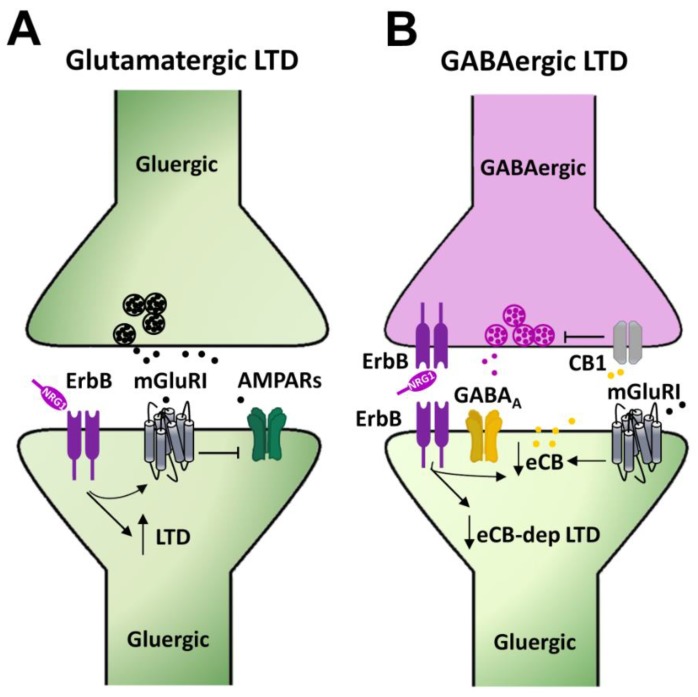
Mechanisms underlying NRGs/ErbB-dependent regulation of mGluRI-LTD in the hippocampus. (**A**) NRG1-dependent activation of ErbB receptors in CA1 pyramidal neurons, by regulating mGluRI function, controls the LTD magnitude of AMPAR-mediated transmission. (**B**) NRG1, by activating ErbB receptors, reduces endocannabinoids (eCBs) levels, thus impairing the eCB-dependent LTD of GABAergic transmission, which is physiologically triggered by the eCB-mediated activation of CB1 located on presynaptic GABAergic terminals, controlling GABA release.

Long term depression (LTD) of glutamatergic synaptic transmission at CA3–CA1 synapses represents a well characterized and largely studied form of synaptic plasticity in the hippocampus. Group 1 metabotropic glutamate receptors (mGluRI), encompassing the mGluR1 and mGluR5 subtypes, are involved in the induction of this form of synaptic plasticity either in the hippocampus or in different other areas, including dorsal and ventral striatum, medial prefrontal cortex, cerebellum, and midbrain DAergic nuclei [87]. mGluRI-LTD at hippocampal CA3–CA1 synapses ultimately relies on mGluRI-induced AMPARs internalization [61,88,89]. Intracellular mechanisms, downstream of mGluRI activation, encompass the activation of several kinases pathways, including ERK1/2, PI3K-Akt-mTOR, and mitogen activated protein kinases (MAPKs) [90,91,92], which fosters the synthesis of “LTD proteins”, instrumental to LTD expression and maintenance [61,87,93]. mGluRI-LTD can be easily achieved through mGluRI activation with the agonist DHPG or by endogenous glutamate, synaptically released through specific electrical protocols, like paired pulse (PP) low frequency stimulation (PP-LFS 1 Hz, 15 min) [94,95,96]. We recently reported that NRG1/ErbB signaling is a critical modulatory pathway of mGluRI-dependent LTD at CA3–CA1 synapses [86]. Actually, NRG1 fosters the induction of mGluRI-LTD in CA1 pyramidal neurons from hippocampal mice slices. Such NRG-dependent facilitation of LTD is especially unmasked in conditions of minimal stimulation of mGluRI, which per se induces only a short term depression of AMPAR-mediated transmission, but causes a stable LTD following NRG1 treatment. More interestingly, preventing endogenous ErbB activation, with ErbB inhibitors, impairs mGluRI-LTD expression. Indeed, the intracellular injection of pan-ErbB inhibitors or selective ErbB2 inhibitors in single CA1 pyramidal cells depresses mGluRI-induced LTD, thus supporting an NRG1/ErbB-dependent cell autonomous mechanism of regulation of mGluRI-mediated synaptic plasticity, involving ErbB2-containing receptors [86] (Figure 4A). Such a postsynaptic model is also supported by the evidence that NRG1/ErbB-dependent effects are observed in the presence of a GABA_A_ antagonist, thus not comprising the interplay of GABAergic transmission, but rather a direct mGluRI regulation in CA1 pyramidal neurons, as similarly occurs in midbrain DA cells (see the section below).

Overall, evidence on the modulatory roles of NRGs/ErbB signaling in glutamatergic synaptic plasticity in the hippocampus indicate that NRG1-dependent ErbB activation dampens glutamatergic LTP and favors mGluRI-dependent glutamatergic LTD at hippocampal CA3–CA1 synapses, thus representing a central mechanism balancing the LTP/LTD equilibrium, which shapes the strength of the excitatory transmission at CA3–CA1 synapses.

#### 3.1.3. mGluRI-dependent GABAergic LTD

While it is largely recognized that NRGs/ErbB signaling plays a prominent role in the regulation of the GABAergic system, affecting synapses development, as well as neurotransmitter release and GABAergic receptors expression/function, evidence regarding long term modifications of GABAergic transmission, by NRGs/ErbB-dependent mechanisms, are scarce.

The activation of mGluRI in hippocampal CA1 pyramidal neurons induces an LTD of GABAergic transmission, which is reliant on mGluRI-induced production of endocannabinoids (eCBs), which act retrogradely on their presynaptic receptors, cannabinoid receptors 1 (CB1Rs), thus inhibiting GABA release [97,98]. Such a form of mGluRI-dependent eCBs-mediated LTD of GABAergic transmission, which is inducible upon mGluRI stimulation, is modulated by NRG1/ErbB signaling [99]. Indeed, a chronic treatment with NRG1 (8–11 days on organotypic hippocampal slices) impairs the expression of mGluRI-dependent GABAergic LTD at CA3–CA1 synapses, via an eCB-dependent mechanism, which is reliant on a reduced tone of eCBs, as a consequence of their increased catabolism [99] (Figure 4B). The intracellular events, by which NRG1-induced ErbB activation potentiates the expression of catabolic enzymes of eCBs, as well as the synaptic compartments where they occur, are still uncharacterized [99]. Notwithstanding, this evidence points to an important role of NRGs/ErbB signaling in the regulation of GABAergic LTD at hippocampal CA3–CA1 synapses, thus adding further to the picture describing the relevance of NRGs/ErbB signaling in the regulation of synaptic plasticity in the hippocampus.

### 3.2. NRGs/ErbB-dependent Regulation of Synaptic Plasticity in Midbrain DA Neurons

Long lasting modifications of synaptic transmission have been reported in midbrain DA nuclei. The evidence is mainly related to the ventral tegmental area (VTA) DA cells, where different forms of synaptic plasticity (LTP and LTD) can be induced by either electrical stimulation protocols or pharmacological activation of mGluRI [100,101,102,103,104,105]. In VTA DA cells, glutamatergic LTP is inducible by HFS protocols paired with neuronal depolarization [100], as well as by a spike-timing-dependent stimulation protocol [106], by mechanisms involving NMDAR activation and increase in intracellular Ca^2+^.

LTD of glutamatergic transmission can be observed in VTA and SNpc DA neurons, upon delivery of LFS (1 Hz, 10 min) paired with neuronal depolarization during stimulation. Such LFS-induced LTD is reliant on the activation of voltage-dependent Ca^2+^ channels, but does not require the activation of NMDAR or glutamatergic metabotropic receptors [101,102]. As occurs in other brain areas, mGluR1 activation triggers LTD of AMPAR-mediated transmission either in VTA [103] or SNpc DA cells [107]. In nigral DA cells, mGluR1-LTD is easily inducible by mGluR1 activation either achieved with an agonist or by synaptic glutamate, endogenously released during electrical LFS [107]. In VTA DA cells from mice, mGluRI-LTD is especially unmasked in already potentiated synapses (e.g., following exposure to psychostimulants) more than in naive synapses [108] and is due to a modification of AMPARs subunit composition, which decreases AMPARs ion conductances [103].

NRGs/ErbB signaling critically regulates glutamatergic synaptic plasticity in SNpc DA neurons [107]. Indeed, ErbB signaling is required to preserve mGluRI-dependent LTD expression in nigral DAergic neurons, the mGluRI-LTD magnitude being reduced by preventing endogenous ErbB stimulation, with ErbB inhibitors that preferentially act on ErbB2/ErbB4 subunits [107]. Furthermore, exogenous NRG1 application fosters mGluRI-dependent LTD in DA neurons, allowing its induction also in the presence of a minimal mGluRI stimulation. Hence, NRG1/ErbB signaling controls the strength of glutamatergic synaptic transmission in midbrain DA neurons, fine tuning the magnitude of LTD of AMPAR-mediated transmission caused by mGluR1 activation [107].

**Figure 5:**
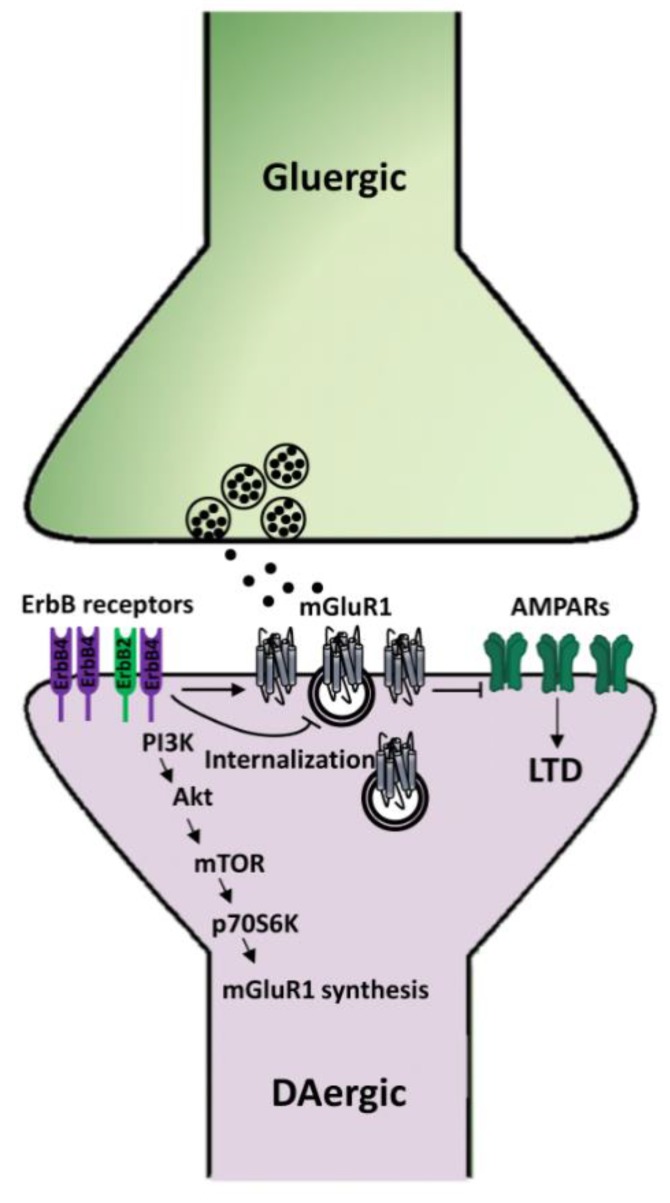
Mechanisms underlying NRG1/ErbB-dependent regulation of mGluRI-LTD in midbrain DA neurons. NRG1-dependent activation of ErbB receptors in SNpc DA neurons induces the synthesis of mGluR1 receptors, thus fostering mGluR1-LTD of AMPARs-mediated transmission, whereas inhibition of endogenous ErbB signaling causes mGluR1 internalization and consequent impairment of mGluR1-LTD.

Regarding the cellular mechanisms underlying NRG1-dependent regulation of mGluRI-LTD, we previously found that NRG1/ErbB signaling bidirectionally controlled mGluR1 expression levels on the surface membrane of midbrain DA neurons [109]. Indeed, NRG1-induced ErbB activation rapidly stimulates mGluR1 synthesis and membrane trafficking. Exogenous NRG1 increases mGluR1 protein levels in SNpc/VTA homogenates and mGluR1 immunolabeling in TH+ DA neurons, in addition to potentiating mGluR1-mediated currents in SNpc DA cells [109]. The mechanisms underlying NRG1-induced increase in mGluR1 expression/function in midbrain DA neurons involved ErbB receptors, possibly as ErbB2/ErbB4 dimers, and downstream activation of PI3K-Akt-mTOR pathways [109]. Importantly, endogenous ErbB signaling controls mGluR1 docking on the membrane surface of midbrain DA neurons, since pharmacological ErbB inhibition causes mGluR1 internalization, which occurs through dynamin-dependent endocytosis. Hence, our evidence suggests that NRG1/ErbB-dependent regulation of mGluRI-LTD in nigral DA neurons is reliant on a direct ErbB-dependent regulation of mGluR1 expression levels, with ErbB receptor activation orchestrating the synthesis, trafficking, and membrane docking of mGluR1 in midbrain DA neurons (Figure 5). Notably, in such a way, NRG1/ErbB signaling controls several mGluR1-dependent functions in SNpc DA neurons, besides mGluRI-LTD, including mGluR1-dependent activation of the nigrostriatal DA pathway in vivo [109], supporting a central role of NRG1/ErbB signaling in the regulation of the midbrain DA system.

## 4. Implications of NRG/ErbB-dependent Regulation of Synaptic Plasticity

What is the physiopathological relevance of NRGs/ErbB-induced modulation of synaptic plasticity? NRGs/ErbB-dependent regulation of the hippocampal synaptic plasticity has obvious implications in learning and memory processes, in which enduring changes of synaptic strength represent the underlying cellular mechanisms. Hippocampal LTP at CA3–CA1 synapses is involved in different learning processes, including contextual fear conditioning. Notably, NRG1/ErbB signaling, besides affecting hippocampal LTP, regulates contextual fear memory, mainly acting on PV+ GABAergic interneurons, as demonstrated by the evidence that a conditional genetic ablation of ErbB4 in this neuronal population impairs this learning process [71]. Contextual fear memory is similarly affected by intra-hippocampal injection of an ErbB inhibitor [110].

mGluRI-dependent LTD at hippocampal CA3–CA1 synapses has been associated with novelty detection and related learning processes, like object recognition memory [87,111]. We recently demonstrated that NRGs/ErbB signaling in the hippocampal CA1 area regulated either mGluRI-dependent LTD or object recognition memory in mice [86]. Indeed, intra-hippocampal injection of an ErbB inhibitor in the CA1 area prevented the induction of mGluRI-LTD and impaired the acquisition of object configurations, affecting the recognition memory [86].

NRG1/ErbB-dependent modulation of synaptic plasticity in the hippocampus has been reported also in pathological contexts, as in animal models of neurological and psychiatric disorders, like Angelman’s syndrome (AS) and Alzheimer’s disease (AD). AS is characterized by autism, mental retardation, motor abnormalities, and epilepsy and is mostly caused by the deletion of portions of the maternal copy of chromosome 15, which includes the UBE3A gene. The AS mouse model, produced by UBE3A deletion, displays hippocampal LTP impairment and deficits in hippocampal-dependent learning processes, like contextual fear memory [112,113], as well as an alteration of NRGs/ErbB signaling, consisting of enhanced levels of NRG1 and ErbB4 phosphorylation in the hippocampus. Interestingly, the injection of an ErbB inhibitor, PD158780, in the dorsal hippocampus, rescues synaptic and behavioral deficits, allowing proper LTP expression at CA3–CA1 synapses and rescuing contextual fear memory [110].

NRGs/ErbB-dependent regulation of hippocampal synaptic plasticity in AD animal models has been also reported. Indeed, NRG1 counteracts amyloid β-induced impairment of LTP in the hippocampal CA1 area and dentate gyrus in mouse slices, through a mechanism involving ErbB4 stimulation [114] and downstream PI3K activation [115]. Moreover, the activation of NRGs/ErbB signaling ameliorates LTP deficits, as well as cognitive abnormalities in adult Tg2576 mice, an AD animal model [116,117]. Overall, this evidence supports a relevant function of NRG1/ErbB-dependent regulation of synaptic plasticity in hippocampal-dependent learning/memory processes and suggests that a modulation of ErbB signaling might represent a rescue strategy for neuropsychiatric disorders, like autism spectrum disorders, intellectual disabilities, and AD, which display abnormalities in hippocampal-related forms of synaptic plasticity.

Conversely, the factual relevance of NRGs/ErbB-dependent regulation of glutamatergic synaptic plasticity in nigral DA neurons is still completely mysterious, mainly because the physiological functions of specific forms of synaptic plasticity in this DA nucleus are yet to be deciphered. However, since it is well recognized that the nigrostriatal DA pathway plays an important role in the establishment of goal-oriented behaviors, like feeding and locomotion, as well as in different cognitive functions, including reward/aversion-based learning, mental flexibility, and habit formation [118,119,120,121,122,123,124], it could be speculated that synaptic plasticity-related mechanisms within SNpc DA cells might contribute and/or underlie these brain processes. In such a perspective, NRGs/ErbB-dependent modulation of mGluRI-dependent LTD might have several implications in the regulation of various cognitive processes and complex behaviors dependent on the midbrain DA system. Regarding a potential link between NRG1/ErbB signaling in midbrain DA neurons and learning processes dependent on mGluR1-dependent synaptic plasticity in SNpc, it should be mentioned that ErbB4 deletion in midbrain DA neurons specifically impairs spatial/working memory [74], which is similarly affected by either systemic administration of mGluR1 antagonists or by a neurotoxin-induced lesion of SNpc [118,125,126,127,128,129]. Therefore, despite a direct connection between nigral ErbB, mGluR1, and working memory is lacking, an interplay between mGluR1-dependent synaptic plasticity and ErbB signaling in this cognitive process related to the nigrostriatal pathway could be conceived. Moreover, mGluR1-LTD in nigral DA neurons might be instrumental to motor learning, in which nigral mGluR1 has been also involved [87,130,131].

Notably, increasing evidence supports the contribution of mGluR1-dependent mechanisms in the pathogenesis of neurological and psychiatric disorders, such as schizophrenia, Parkinson’s disease (PD), addiction, and autism [87,132,133,134], which are either characterized by alterations in midbrain DA transmission and also linked to NRG1/ErbB dysfunctions [13,46,135,136,137]. Whether or not a dysfunction in NRGs/ErbB-dependent regulation of synaptic plasticity in midbrain DAergic neurons represents a factual contributing factor in these pathological conditions is still to be ascertained. Nevertheless, genetic evidence suggests an association between altered NRG1/ErbB signaling and drug of abuse dependence [135,136,137,138] or autism [136,139], and indeed, dysregulated mGluRI-LTD in VTA DA neurons is instrumental to the establishment of addiction-related behaviors [87] and has been reported in an animal model of autism [140]. Future studies might unveil if altered NRGs/ErbB-dependent regulation of mGluR1-LTD in midbrain DA neurons contributes to the aberrant DA transmission occurring in schizophrenia, which is the first neuropsychiatric disorder in which the alteration of NRGs/ErbB signaling has been overtly shown [13].

## 5. Conclusions and Outstanding Issues

Growing evidence indicates that NRGs/ErbB signaling is essential for proper brain development and functioning. While NRGs have multifaceted roles in neurodevelopment, controlling synapse formation and myelination, the modulation of synaptic plasticity in adult brain probably represents the prominent mechanism by which NRGs can affect cognitive functions and complex behaviors in adulthood. The mechanisms engaged by NRGs in the regulation of synaptic plasticity are various. They occur at either presynaptic or postsynaptic compartments and involve modifications of neurotransmitters release or direct modulation of neurotransmitter receptors, which are chief players in the induction and/or expression of different forms of synaptic plasticity, like NMDAR, AMPAR, and mGluRI.

In the hippocampus, NRGs-dependent ErbB activation impairs LTP induction and causes depotentiation at CA3–CA1 synapses. The mechanisms underlying such LTP impairment involve NRGs-dependent enhancement of GABAergic transmission, regulation of DA extracellular levels, and postsynaptic NMDARs inhibition. At hippocampal CA3–CA1 synapses, NRGs/ErbB signaling also fosters mGluRI-dependent LTD, by preserving mGluRI function, thus shifting LTP/LTD equilibrium toward inhibition of glutamatergic transmission. Moreover, NRGs-induced ErbB activation impairs mGluRI/eCB-dependent GABAergic LTD at CA3–CA1 synapses, implying that NRGs have multifaceted roles in the long term regulation of excitatory/inhibitory balance in the hippocampus.

In midbrain DA neurons, NRGs/ErbB receptors are essential for the induction of mGluRI-dependent glutamatergic LTD. Indeed, NRGs/ErbB signaling favors mGluR1 synthesis, trafficking, and membrane docking in DA neurons, thus affecting mGluRI-LTD magnitude and midbrain DA system activation.

Although, herein, the description of NRGs/ErbB-dependent roles in the modulation of synaptic plasticity has been focused on the hippocampus and midbrain DA nuclei, it should be considered that emerging evidence also documented NRGs-induced regulation of synaptic plasticity in other brain areas, and several reports mainly obtained in transgenic mice with altered NRGs/ErbB signaling support that the proper NRGs/ErbB tone (neither too much nor too little) is essential for various cognitive functions and behaviors. Since dysfunctions in NRGs/ErbB signaling have been linked to different neurological and psychiatric disorders, including schizophrenia, bipolar disorder, autism spectrum disorders, genetic intellectual disabilities, AD, major depressive disorder, PD, and addiction [23,46,136,137,138,139,141,142,143,144,145,146,147,148,149,150,151,152,153,154,155,156,157,158,159], intense research efforts should be aimed at deciphering NRGs-dependent mechanisms causing pathological defects, as this might have noticeable implications on the understanding and treatment of different serious brain diseases.

## References

[1] Holmes W.E., Sliwkowski M.X., Akita R.W., Henzel W.J., Lee J., Park J.W., Yansura D., Abadi N., Raab H., Lewis G.D. (1992). Identification of Heregulin, a Specific Activator of p185erbB2. Science.

[2] Peles E., Bacus S.S., Koski R.A., Lu H.S., Wen D., Ogden S.G., Levy R.B., Yarden Y. (1992). Isolation of the neu/HER-2 stimulatory ligand: A 44 kd glycoprotein that induces differentiation of mammary tumor cells. Cell.

[3] Wen D., Peles E., Cupples R., Suggs S.V., Bacus S.S., Luo Y., Trail G., Hu S., Silbiger S.M., Levy R.B. (1992). Neu differentiation factor: A transmembrane glycoprotein containing an EGF domain and an immunoglobulin homology unit. Cell.

[4] Raff M.C., Abney E., Brockes J.P., Hornby-Smith A. (1978). Schwann cell growth factors. Cell.

[5] Brockes J.P., Lemke G.E., Balzer D.R. (1980). Purification and preliminary characterization of a glial growth factor from the bovine pituitary. J. Biol. Chem..

[6] Lemke G., Brockes J.P. (1984). Identification and purification of glial growth factor. J. Neurosci..

[7] Goodearl A.D., Davis J.B., Mistry K., Minghetti L., Otsu M., Waterfield M.D., Stroobant P. (1993). Purification of multiple forms of glial growth factor. J. Biol. Chem..

[8] Marchionni M.A., Goodearl A.D., Chen M.S., Bermingham-McDonogh O., Kirk C., Hendricks M., Danehy F., Misumi D., Sudhalter J., Kobayashi K. (1993). Glial growth factors are alternatively spliced erbB2 ligands expressed in the nervous system. Nature.

[9] Falls D.L., Rosen K.M., Corfas G., Lane W.S., Fischbach G.D. (1993). ARIA, a protein that stimulates acetylcholine receptor synthesis, is a member of the neu ligand family. Cell.

[10] Brown N.J., Holguin B., Lin B. (2004). Expression of Neuregulin 1, a Member of the Epidermal Growth Factor Family, Is Expressed as Multiple Splice Variants in the Adult Human Cornea. Investig. Opthalmol. Vis. Sci..

[11] Steinthorsdottir V., Stefansson H., Ghosh S., Birgisdottir B., Bjornsdottir S., Fasquel A.C., Olafsson O., Stefansson K., Gulcher J.R. (2004). Multiple novel transcription initiation sites for NRG1. Gene.

[12] Tan W., Wang Y., Gold B., Chen J., Dean M., Harrison P.J., Weinberger D.R., Law A.J. (2007). Molecular Cloning of a Brain-specific, Developmentally Regulated Neuregulin 1 (NRG1) Isoform and Identification of a Functional Promoter Variant Associated with Schizophrenia. J. Biol. Chem..

[13] Mei L., Xiong W.C. (2008). Neuregulin 1 in neural development, synaptic plasticity and schizophrenia. Nat. Rev. Neurosci..

[14] Liu X., Bates R., Yin D.M., Shen C., Wang F., Su N., Kirov S.A., Luo Y., Wang J.Z., Xiong W.C. (2011). Specific Regulation of NRG1 Isoform Expression by Neuronal Activity. J. Neurosci..

[15] Li Q., Loeb J.A. (2001). Neuregulin-heparan-sulfate proteoglycan interactions produce sustained erbB receptor activation required for the induction of acetylcholine receptors in muscle. J. Biol. Chem..

[16] Ho W.-H., Armanini M.P., Nuijens A., Phillips H.S., Osheroff P.L. (1995). Sensory and Motor Neuron-derived Factor.A novel heregulin variant highly expressed in sensory and motor neurons. J. Biol. Chem..

[17] Law A.J., Shannon Weickert C., Hyde T.M., Kleinman J.E., Harrison P.J. (2004). Neuregulin-1 (NRG-1) mRNA and protein in the adult human brain. Neuroscience.

[18] Chang H., Riese D.J., Gilbert W., Stern D.F., McMahan U.J. (1997). Ligands for ErbB-family receptors encoded by a neuregulin-like gene. Nature.

[19] Carraway K.L., Weber J.L., Unger M.J., Ledesma J., Yu N., Gassmann M., Lai C. (1997). Neuregulin-2, a new ligand of ErbB3/ErbB4-receptor tyrosine kinases. Nature.

[20] Busfield S.J., Michnick D.A., Chickering T.W., Revett T.L., Ma J., Woolf E.A., Comrack C.A., Dussault J., Woolf J., Goodearl A.D. (1997). Characterization of a neuregulin-related gene, Don-1, that is highly expressed in restricted regions of the cerebellum and hippocampus. Mol. Cell. Biol..

[21] Yan L., Shamir A., Skirzewski M., Leiva-Salcedo E., Kwon O.B., Karavanova I., Paredes D., Malkesman O., Bailey K.R., Vullhorst D. (2018). Neuregulin-2 ablation results in dopamine dysregulation and severe behavioral phenotypes relevant to psychiatric disorders. Mol. Psychiatry.

[22] Carteron C., Ferrer-Montiel A., Cabedo H. (2006). Characterization of a neural-specific splicing form of the human neuregulin 3 gene involved in oligodendrocyte survival. J. Cell. Sci..

[23] Kao W.T., Wang Y., Kleinman J.E., Lipska B.K., Hyde T.M., Weinberger D.R., Law A.J. (2010). Common genetic variation in Neuregulin 3 (NRG3) influences risk for schizophrenia and impacts NRG3 expression in human brain. Proc. Natl. Acad. Sci. USA.

[24] Paterson C., Wang Y., Hyde T.M., Weinberger D.R., Kleinman J.E., Law A.J. (2017). Temporal, Diagnostic, and Tissue-Specific Regulation of NRG3 Isoform Expression in Human Brain Development and Affective Disorders. Am. J. Psychiatry.

[25] Zeledón M., Eckart N., Taub M., Vernon H., Szymanksi M., Wang R., Chen P.L., Nestadt G., McGrath J.A., Sawa A. (2015). Identification and functional studies of regulatory variants responsible for the association of *NRG3* with a delusion phenotype in schizophrenia. Mol. Neuropsychiatry.

[26] Zhang D., Sliwkowski M.X., Mark M., Frantz G., Akita R., Sun Y., Hillan K., Crowley C., Brush J., Godowski P.J. (1997). Neuregulin-3 (NRG3): A novel neural tissue-enriched protein that binds and activates ErbB4. Proc. Natl. Acad. Sci. USA.

[27] Harari D., Tzahar E., Romano J., Shelly M., Pierce J.H., Andrews G.C., Yarden Y. (1999). Neuregulin-4: A novel growth factor that acts through the ErbB-4 receptor tyrosine kinase. Oncogene.

[28] Hayes N.V., Gullick W.J. (2008). The neuregulin family of genes and their multiple splice variants in breast cancer. J. Mammary Gland Biol. Neoplasia.

[29] Rosell M., Kaforou M., Frontini A., Okolo A., Chan Y.W., Nikolopoulou E., Millership S., Fenech M.E., MacIntyre D., Turner J.O. (2014). Brown and white adipose tissues: Intrinsic differences in gene expression and response to cold exposure in mice. Am. J. Physiol. Endocrinol. Metab..

[30] Paramo B., Wyatt S., Davies A.M. (2018). An essential role for neuregulin-4 in the growth and elaboration of developing neocortical pyramidal dendrites. Exp. Neurol..

[31] Uchida T., Wada K., Akamatsu T., Yonezawa M., Noguchi H., Mizoguchi A., Kasuga M., Sakamoto C. (1999). A novel epidermal growth factor-like molecule containing two follistatin modules stimulates tyrosine phosphorylation of erbB-4 in MKN28 gastric cancer cells. Biochem. Biophys. Res. Commun..

[32] Mei L., Nave K.A. (2014). Neuregulin-ERBB signaling in the nervous system and neuropsychiatric diseases. Neuron.

[33] Kanemoto N., Horie M., Omori K., Nishino N., Kondo M., Noguchi K., Tanigami A. (2001). Expression of TMEFF1 mRNA in the mouse central nervous system: Precise examination and comparative studies of TMEFF1 and TMEFF2. Brain Res. Mol. Brain Res..

[34] Kinugasa Y., Ishiguro H., Tokita Y., Oohira A., Ohmoto H., Higashiyama S. (2004). Neuroglycan C, a novel member of the neuregulin family. Biochem. Biophys. Res. Commun..

[35] Aono S., Tokita Y., Yasuda Y., Hirano K., Yamauchi S., Shuo T., Matsui F., Keino H., Kashiwai A., Kawamura N. (2006). Expression and identification of a new splice variant of neuroglycan C, a transmembrane chondroitin sulfate proteoglycan, in the human brain. J. Neurosci. Res..

[36] Falls D.L. (2003). Neuregulins: Functions, forms, and signaling strategies. Exp. Cell. Res..

[37] Vullhorst D., Ahmad T., Karavanova I., Keating C., Buonanno A. (2017). Structural Similarities between Neuregulin 1-3 Isoforms Determine Their Subcellular Distribution and Signaling Mode in Central Neurons. J. Neurosci..

[38] Eilam R., Pinkas-Kramarski R., Ratzkin B.J., Segal M., Yarden Y. (1998). Activity dependent regulation of Neu differentiation factor/neuregulin expression in rat brain. Proc. Natl. Acad. Sci. USA.

[39] Arora P., Cuevas B.D., Russo A., Johnson G.L., Trejo J. (2008). Persistent transactivation of EGFR and ErbB2/HER2 by protease-activated receptor-1 promotes breast carcinoma cell invasion. Oncogene.

[40] Li Q., Zhang R., Ge Y.L., Mei Y.W., Guo Y.L. (2009). Effects of neuregulin on expression of MMP-9 and NSE in brain of ischemia/reperfusion rat. J. Mol. Neurosci..

[41] Yarden Y., Sliwkowski M.X. (2001). Untangling the ErbB signalling network. Nat. Rev. Mol. Cell. Biol..

[42] Graus-Porta D., Beerli R.R., Daly J.M., Hynes N.E. (1997). ErbB-2, the preferred heterodimerization partner of all ErbB receptors, is a mediator of lateral signaling. EMBO J..

[43] Sliwkowski M.X., Schaefer G., Akita R.W., Lofgren A., Fitzpatrick V.D., Nuijens A., Fendly B.M., Cerione R.A., Vandlen R.L., Carraway K.L. (1994). Coexpression of erbB2 and erbB3 proteins reconstitutes a high affinity receptor for heregulin. J. Biol. Chem..

[44] Wang L.M., Kuo A., Alimandi M., Veri M.C., Lee C.C., Kapoor V., Ellmore N., Chen X.H., Pierce J.H. (1998). ErbB2 expression increases the spectrum and potency of ligand mediated signal transduction through ErbB4. Proc. Natl. Acad. Sci. USA.

[45] Jones J.T., Ballinger M.D., Pisacane P.I., Lofgren J.A., Fitzpatrick V.D., Fairbrother W.J., Wells J.A., Sliwkowski M.X. (1998). Binding interaction of the heregulinbeta egf domain with ErbB3 and ErbB4 receptors assessed by alanine scanning mutagenesis. J. Biol. Chem..

[46] Iwakura Y., Nawa H. (2013). ErbB1–4 dependent EGF/neuregulin signals and their cross talk in the central nervous system: Pathological implications in schizophrenia and Parkinson’s disease. Front. Cell. Neurosci..

[47] Kim J.H., Saito K., Yokoyama S. (2002). Chimeric receptor analyses of the interactions of the ectodomains of ErbB-1 with epidermal growth factor and of those of ErbB-4 with neuregulin. Eur. J. Biochem..

[48] Olayioye M.A., Graus-Porta D., Beerli R.R., Rohrer J., Gay B., Hynes N.E. (1998). ErbB-1 and ErbB-2 acquire distinct signaling properties dependent upon their dimerization partner. Mol. Cell. Biol..

[49] Iwakura Y., Piao Y.S., Mizuno M., Takei N., Kakita A., Takahashi H., Nawa H. (2005). Influences of dopaminergic lesion on epidermal growth factor-ErbB signals in Parkinson’s disease and its model: Neurotrophic implication in nigrostriatal neurons. J. Neurochem..

[50] Nagano T., Namba H., Abe Y., Aoki H., Takei N., Nawa H. (2007). In vivo administration of epidermal growth factor and its homologue attenuates developmental maturation of functional excitatory synapses in cortical GABAergic neurons. Eur. J. Neurosci..

[51] Namba H., Zheng Y., Abe Y., Nawa H. (2009). Epidermal growth factor administered in the periphery influences excitatory synaptic inputs onto midbrain dopaminergic neurons in postnatal mice. Neuroscience.

[52] Bjarnadottir M., Misner D.L., Haverfield-Gross S., Bruun S., Helgason V.G., Stefansson H., Sigmundsson A., Firth D.R., Nielsen B., Stefansdottir R. (2007). Neuregulin 1 (NRG1) signaling through Fyn modulates NMDA receptor phosphorylation: Differential synaptic function in NRG1C knock-outs compared with wild-type mice. J. Neurosci..

[53] Fu A.K., Fu W.Y., Cheung J., Tsim K.W., Ip F.C., Wang J.H., Ip N.Y. (2001). Cdk5 is involved in neuregulin induced AChR expression at the neuromuscular junction. Nat. Neurosci..

[54] Si J., Mei L. (1999). ERK MAP kinase activation is required for acetylcholine receptor inducing activity induced increase in all five acetylcholine receptor subunit mRNAs as well as synapse-specific expression of acetylcholine receptor epsilon-transgene. Brain Res. Mol. Brain Res..

[55] Hellyer N.J., Cheng K., Koland J.G. (1998). ErbB3 (HER3) interaction with the p85 regulatory subunit of phosphoinositide 3-kinase. J. Biochem..

[56] Lee H.J., Jung K.M., Huang Y.Z., Bennett L.B., Lee J.S., Mei L., Kim T.W. (2002). Presenilin dependent gamma-secretase-like intramembrane cleavage of ErbB4. J. Biol. Chem..

[57] Ni C.Y., Murphy M.P., Golde T.E., Carpenter G. (2001). gamma -Secretase cleavage and nuclear localization of ErbB-4 receptor tyrosine kinase. Science.

[58] Sardi S.P., Murtie J., Koirala S., Patten B.A., Corfas G. (2006). Presenilin dependent ErbB4 nuclear signaling regulates the timing of astrogenesis in the developing brain. Cell.

[59] Bao J., Wolpowitz D., Role L.W., Talmage D.A. (2003). Back signaling by the Nrg-1 intracellular domain. J. Cell. Biol..

[60] Bao J., Lin H., Ouyang Y., Lei D., Osman A., Kim T.-W., Mei L., Dai P., Ohlemiller K.K., Ambron R.T. (2004). Activity-dependent transcription regulation of PSD-95 by neuregulin-1 and Eos. Nat. Neurosci..

[61] Malenka R.C., Bear M.F. (2004). LTP and LTD: An embarrassment of riches. Neuron.

[62] Kandel E.R., Dudai Y., Mayford M.R. (2014). The molecular and systems biology of memory. Cell.

[63] Nicoll R.A. (2017). A Brief History of Long-Term Potentiation. Neuron.

[64] Collingridge G.L., Peineau S., Howland J.G., Wang Y.T. (2010). Long-term depression in the CNS. Nat. Rev. Neurosci..

[65] Dan Y., Poo M.M. (2006). Spike timing dependent plasticity: From synapse to perception. Physiol. Rev..

[66] Markram H., Lübke J., Frotscher M., Sakmann B. (1997). Regulation of synaptic efficacy by coincidence of postsynaptic APs and EPSPs. Science.

[67] Larson J., Wong D., Lynch G. (1986). Patterned stimulation at the theta frequency is optimal for the induction of hippocampal long-term potentiation. Brain Res..

[68] Hölscher C., Anwyl R., Rowan M.J. (1997). Stimulation on the Positive Phase of Hippocampal Theta Rhythm Induces Long-Term Potentiation That Can Be Depotentiated by Stimulation on the Negative Phase in Area CA1 In Vivo. J. Neurosci..

[69] Huang Y.Z., Won S., Ali D.W., Wang Q., Tanowitz M., Du Q.S., Pelkey K.A., Yang D.J., Xiong W.C., Salter M.W. (2000). Regulation of neuregulin signaling by PSD-95 interacting with ErbB4 at CNS synapses. Neuron.

[70] Kwon O.B., Longart M., Vullhorst D., Hoffman D.A., Buonanno A. (2005). Neuregulin- 1 reverses long-term potentiation at CA1 hippocampal synapses. J. Neurosci..

[71] Chen Y.J., Zhang M., Yin D.M., Wen L., Ting A., Wang P., Lu Y.S., Zhu X.H., Li S.J., Wu C.Y. (2010). ErbB4 in parvalbumin-positive interneurons is critical for neuregulin 1 regulation of long-term potentiation. Proc. Natl. Acad. Sci. USA.

[72] Pitcher G.M., Kalia L.V., Ng D., Goodfellow N.M., Yee K.T., Lambe E.K., Salter M.W. (2011). Schizophrenia susceptibility pathway Neuregulin 1-ErbB4 suppresses Src upregulation of NMDA receptors. Nat. Med..

[73] Kwon O.B., Paredes D., Gonzalez C.M., Neddens J., Hernandez L., Vullhorst D., Buonanno A. (2008). Neuregulin-1 regulates LTP at CA1 hippocampal synapses through activation of dopamine D4 receptors. Proc. Natl. Acad. Sci. USA.

[74] Skirzewski M., Karavanova I., Shamir A., Erben L., Garcia-Olivares J., Shin J.H., Vullhorst D., Alvarez V.A., Amara S.G., Buonanno A. (2018). ErbB4 signaling in dopaminergic axonal projections increases extracellular dopamine levels and regulates spatial/working memory behaviors. Mol. Psychiatry.

[75] Izumi Y., Zorumski C.F. (2017). Neuregulin and Dopamine D4 Receptors Contribute Independently to Depotentiation of Schaffer Collateral LTP by Temperoammonic Path Stimulation. eNeuro.

[76] Vullhorst D., Neddens J., Karavanova I., Tricoire L., Petralia R.S., McBain C.J., Buonanno A. (2009). Selective expression of ErbB4 in interneurons, but not pyramidal cells, of the rodent hippocampus. J. Neurosci..

[77] Madisen L., Zwingman T.A., Sunkin S.M., Oh S.W., Zariwala H.A., Gu H., Ng L.L., Palmiter R.D., Hawrylycz M.J., Jones A.R. (2010). A robust and high-throughput Cre reporting and characterization system for the whole mouse brain. Nat. Neurosci..

[78] Bean J.C., Lin T.W., Sathyamurthy A., Liu F., Yin D.M., Xiong W.C., Mei L. (2014). Genetic labeling reveals novel cellular targets of schizophrenia susceptibility gene: Distribution of GABA and non-GABA ErbB4-positive cells in adult mouse brain. J. Neurosci..

[79] Gerecke K.M., Wyss J.M., Karavanova I., Buonanno A., Carroll S.L. (2001). ErbB transmembrane tyrosine kinase receptors are differentially expressed throughout the adult rat central nervous system. J. Comp. Neurol..

[80] Thompson M., Lauderdale S., Webster M.J., Chong V.Z., McClintock B., Saunders R., Weickert C.S. (2007). Widespread expression of ErbB2, ErbB3 and ErbB4 in non-human primate brain. Brain Res..

[81] Lai C., Lemke G. (1991). An extended family of protein-tyrosine kinase genes differentially expressed in the vertebrate nervous system. Neuron.

[82] Mechawar N., Lacoste B., Yu W.F., Srivastava L.K., Quirion R. (2007). Developmental profile of neuregulin receptor ErbB4 in postnatal rat cerebral cortex and hippocampus. Neuroscience.

[83] Li B., Woo R.S., Mei L., Malinow R. (2007). The neuregulin-1 receptor erbB4 controls glutamatergic synapse maturation and plasticity. Neuron.

[84] Barros C.S., Calabrese B., Chamero P., Roberts A.J., Korzus E., Lloyd K., Stowers L., Mayford M., Halpain S., Müller U. (2009). Impaired maturation of dendritic spines without disorganization of cortical cell layers in mice lacking NRG1/ErbB signaling in the central nervous system. Proc. Natl. Acad. Sci. USA.

[85] Cooper M.A., Koleske A.J. (2014). Ablation of ErbB4 from excitatory neurons leads to reduced dendritic spine density in mouse prefrontal cortex. J. Comp. Neurol..

[86] Ledonne A., Mango D., Latagliata E.C., Chiacchierini G., Nobili A., Nisticò R., D’Amelio M., Puglisi-Allegra S., Mercuri N.B. (2018). Neuregulin 1/ErbB signalling modulates hippocampal mGluRI dependent LTD and object recognition memory. Pharmacol. Res..

[87] Lüscher C., Huber K.M. (2010). Group 1 mGluR dependent synaptic long-term depression: Mechanisms and implications for circuitry and disease. Neuron.

[88] Snyder E.M., Philpot B.D., Huber K.M., Dong X., Fallon J.R., Bear M.F. (2001). Internalization of ionotropic glutamate receptors in response to mGluR activation. Nat. Neurosci..

[89] Xiao M.Y., Zhou Q., Nicoll R.A. (2001). Metabotropic glutamate receptor activation causes a rapid redistribution of AMPA receptors. Neuropharmacology.

[90] Thiels E., Kanterewicz B.I., Norman E.D., Trzaskos J.M., Klann E. (2002). Long-term depression in the adult hippocampus in vivo involves activation of extracellular signal-regulated kinase and phosphorylation of Elk-1. J. Neurosci..

[91] Gallagher S.M., Daly C.A., Bear M.F., Huber K.M. (2004). Extracellular signal-regulated protein kinase activation is required for metabotropic glutamate receptor dependent long-term depression in hippocampal area CA1. J. Neurosci..

[92] Hou L., Klann E. (2004). Activation of the phosphoinositide 3-kinase-Akt-mammalian target of rapamycin signaling pathway is required for metabotropic glutamate receptor dependent long-term depression. J. Neurosci..

[93] Huber K.M., Kayser M.S., Bear M.F. (2000). Role for rapid dendritic protein synthesis in hippocampal mGluR dependent long-term depression. Science.

[94] Kemp N., Bashir Z.I. (1999). Induction of LTD in the adult hippocampus by the synaptic activation of AMPA/kainate and metabotropic glutamate receptors. Neuropharmacology.

[95] Huber K.M., Roder J.C., Bear M.F. (2001). Chemical induction of mGluR5- and protein synthesis- dependent long-term depression in hippocampal area CA1. J. Neurophysiol..

[96] Volk L.J., Daly C.A., Huber K.M. (2006). Differential roles for group 1 mGluR subtypes in induction and expression of chemically induced hippocampal long-term depression. J. Neurophysiol..

[97] Chevaleyre V., Castillo P.E. (2003). Heterosynaptic LTD of hippocampal GABAergic synapses: A novel role of endocannabinoids in regulating excitability. Neuron.

[98] Castillo P.E., Younts T.J., Chávez A.E., Hashimotodani Y. (2012). Endocannabinoid signaling and synaptic function. Neuron.

[99] Du H., Kwon I.K., Kim J. (2013). Neuregulin-1 impairs the long-term depression of hippocampal inhibitory synapses by facilitating the degradation of endocannabinoid 2-AG. J. Neurosci..

[100] Bonci A., Malenka R.C. (1999). Properties and plasticity of excitatory synapses on dopaminergic and GABAergic cells in the ventral tegmental area. J. Neurosci..

[101] Jones S., Kornblum J.L., Kauer J.A. (2000). Amphetamine blocks long-term synaptic depression in the ventral tegmental area. J. Neurosci..

[102] Thomas M.J., Malenka R.C., Bonci A. (2000). Modulation of long-term depression by dopamine in the mesolimbic system. J. Neurosci..

[103] Bellone C., Lüscher C. (2005). mGluRs induce a long-term depression in the ventral tegmental area that involves a switch of the subunit composition of AMPA receptors. Eur. J. Neurosci..

[104] Liu Q.S., Pu L., Poo M.M. (2005). Repeated cocaine exposure in vivo facilitates LTP induction in midbrain dopamine neurons. Nature.

[105] Luu P., Malenka R.C. (2008). Spike timing dependent long-term potentiation in ventral tegmental area dopamine cells requires PKC. J. Neurophysiol..

[106] Feldman D.E. (2012). The spike-timing dependence of plasticity. Neuron.

[107] Ledonne A., Mercuri N.B. (2018). mGluR1-Dependent Long Term Depression in Rodent Midbrain Dopamine Neurons Is Regulated by Neuregulin 1/ErbB Signaling. Front. Mol. Neurosci..

[108] Mameli M., Balland B., Luján R., Lüscher C. (2007). Rapid synthesis and synaptic insertion of GluR2 for mGluR-LTD in the ventral tegmental area. Science.

[109] Ledonne A., Nobili A., Latagliata E.C., Cavallucci V., Guatteo E., Puglisi-Allegra S., D’Amelio M., Mercuri N.B. (2015). Neuregulin 1 signalling modulates mGluR1 function in mesencephalic dopaminergic neurons. Mol. Psychiatry.

[110] Kaphzan H., Hernandez P., In Jung J., Cowansage K.K., Deinhardt K., Chao M.V., Abel T., Klann E. (2012). Reversal of Impaired Hippocampal Long-Term Potentiation and Contextual Fear Memory Deficits in Angelman Syndrome Model Mice by ErbB Inhibitors. Biol. Psychiatry.

[111] Goh J.J., Manahan-Vaughan D. (2013). Endogenous hippocampal LTD that is enabled by spatial object recognition requires activation of NMDA receptors and the metabotropic glutamate receptor, mGlu5. Hippocampus.

[112] Jiang Y.H., Armstrong D., Albrecht U., Atkins C.M., Noebels J.L., Eichele G. (1998). Mutation of the Angelman ubiquitin ligase in mice causes increased cytoplasmic p53 and deficits of contextual learning and long term potentiation. Neuron.

[113] Van Woerden G.M., Harris K.D., Hojjati M.R., Gustin R.M., Qiu S., de Avila Freire R., Jiang Y.H., Elgersma Y., Weeber E.J. (2007). Rescue of neurological deficits in a mouse model for Angelman syndrome by reduction of alphaCaMKII inhibitory phosphorylation. Nat. Neurosci..

[114] Min S.S., An J., Lee J.H., Seol G.H., Im J.H., Kim H.S., Baik T.S., Woo R.S. (2011). Neuregulin-1 prevents amyloid beta induced impairment of long-term potentiation in hippocampal slices via ErbB4. Neurosci. Lett..

[115] Baik T.K., Kim Y.-J., Kang S.-M., Song D.-Y., Min S.S., Woo R.-S. (2016). Blocking the phosphatidylinositol 3-kinase pathway inhibits neuregulin-1 mediated rescue of neurotoxicity induced by Ab1–42. J. Pharm. Pharmacol..

[116] Xu J., De Winter F., Farrokhi C., Rockenstein E., Mante M., Adame A., Cook J., Jin X., Masliah E., Lee K.-F. (2016). Neuregulin 1 improves cognitive deficits and neuropathology in an Alzheimer’s disease model. Sci. Rep..

[117] Ryu J., Hong B.H., Kim Y.J., Yang E.-J., Choi M., Kim H., Ahn S., Baik T.-K., Woo R.-S., Kim H.-S. (2016). Neuregulin-1 attenuates cognitive function impairments in a transgenic mouse model of Alzheimer’s disease. Cell Death Dis..

[118] Da Cunha C., Angelucci M.E.M., Canteras N.S., Wonnacott S., Takahashi R.N. (2002). The lesion of the rat substantia nigra pars compacta dopaminergic neurons as a model for Parkinson’s disease memory disabilities. Cell. Mol. Neurobiol..

[119] Da Cunha C., Silva M.H.C., Wietzikoski S., Wietzikoski E.C., Ferro M.M., Kouzmine I., Canteras N.S. (2006). Place learning strategy of substantia nigra pars compacta-lesioned rats. Behav. Neurosci..

[120] Palmiter R.D. (2008). Dopamine signaling in the dorsal striatum is essential for motivated behaviors: Lessons from dopamine-deficient mice. Ann. N. Y. Acad. Sci..

[121] Wise R.A. (2009). Roles for nigrostriatal—Not just mesocorticolimbic—Dopamine in reward and addiction. Trends Neurosci..

[122] Haber S.N. (2014). The place of dopamine in the cortico-basal ganglia circuit. Neuroscience.

[123] Ilango A., Kesner A.J., Keller K.L., Stuber G.D., Bonci A., Ikemoto S. (2014). Similar roles of substantia nigra and ventral tegmental dopamine neurons in reward and aversion. J. Neurosci..

[124] Ledonne A., Mercuri N.B. (2017). Current Concepts on the Physiopathological Relevance of Dopaminergic Receptors. Front. Cell. Neurosci..

[125] Da Cunha C., Wietzikoski S., Wietzikoski E.C., Miyoshi E., Ferro M.M., Anselmo-Franci J.A., Canteras N.S. (2003). Evidence for the substantia nigra pars compacta as an essential component of a memory system independent of the hippocampal memory system. Neurobiol. Learn. Mem..

[126] Miyoshi E., Wietzikoski S., Camplessei M., Silveira R., Takahashi R.N., Da Cunha C. (2002). Impaired learning in a spatial working memory version and in a cued version of the water maze in rats with MPTP induced mesencephalic dopaminergic lesions. Brain Res. Bull..

[127] Braga R., Kouzmine I., Canteras N.S., Da Cunha C. (2005). Lesion of the substantia nigra pars compacta impairs delayed alternation in a Y-maze in rats. Exp. Neurol..

[128] Hsieh M.H., Ho S.C., Yeh K.Y., Pawlak C.R., Chang H.M., Ho Y.J., Lai T.-J., Wu F.-Y. (2010). Blockade of metabotropic glutamate receptors inhibits cognition and neurodegeneration in an MPTP induced Parkinson’s disease rat model. Pharmacol. Biochem. Behav..

[129] Sy H.N., Wu S.L., Wang W.F., Chen C.H., Huang Y.T., Liou Y.M., Chiou C.-S., Pawlak C.R., Ho Y.-J. (2010). MPTP induced dopaminergic degeneration and deficits in object recognition in rats are accompanied by neuroinflammation in the hippocampus. Pharmacol. Biochem. Behav..

[130] Conn P.J., Battaglia G., Marino M.J., Nicoletti F. (2005). Metabotropic glutamate receptors in the basal ganglia motor circuit. Nat. Rev. Neurosci..

[131] Hodgson R.A., Hyde L.A., Guthrie D.H., Cohen-Williams M.E., Leach P.T., Kazdoba T.M., Bleickardt C.J., Lu S.X., Parker E.M., Varty G.B. (2011). Characterization of the selective mGluR1 antagonist, JNJ16259685, in rodent models of movement and coordination. Pharmacol. Biochem. Behav..

[132] Ferraguti F., Crepaldi L., Nicoletti F. (2008). Metabotropic glutamate 1 receptor: Current concepts and perspectives. Pharmacol. Rev..

[133] Lesage A., Steckler T. (2010). Metabotropic glutamate mGlu1 receptor stimulation and blockade: Therapeutic opportunities in psychiatric illness. Eur. J. Pharmacol..

[134] Herman E.J., Bubser M., Conn P.J., Jones C.K. (2012). Metabotropic glutamate receptors for new treatments in schizophrenia. Handb. Exp. Pharmacol..

[135] Han S., Yang B.Z., Kranzler H.R., Oslin D., Anton R., Farrer L.A., Gelernter J. (2012). Linkage analysis followed by association show NRG1 associated with cannabis dependence in African Americans. Biol. Psychiatry.

[136] Yoo H.J., Woo R.S., Cho S.C., Kim B.N., Kim J.W., Shin M.S., Park T.W., Son J.W., Chung U.S., Park S. (2015). Genetic association analyses of neuregulin 1 gene polymorphism with endopheontype for sociality of Korean autism spectrum disorders family. Psychiatry Res..

[137] Ikawa D., Makinodan M., Iwata K., Ohgidani M., Kato T.A., Yamashita Y., Yamamuro K., Kimoto S., Toritsuka M., Yamauchi T. (2017). Microglia-derived neuregulin expression in psychiatric disorders. Brain Behav. Immun..

[138] Turner J.R., Ray R., Lee B., Everett L., Xiang J., Jepson C., Kaestner K.H., Lerman C., Blendy J.A. (2014). Evidence from mouse and man for a role of neuregulin 3 in nicotine dependence. Mol. Psychiatry.

[139] Pinto D., Pagnamenta A.T., Klei L., Anney R., Merico D., Regan R., Conroy J., Magalhaes T.R., Correia C., Abrahams B.S. (2010). Functional impact of global rare copy number variation in autism spectrum disorders. Nature.

[140] Bariselli S., Tzanoulinou S., Glangetas C., Prévost-Solié C., Pucci L., Viguié J., Bezzi P., O’Connor E.C., Georges F., Lüscher C. (2016). SHANK3 controls maturation of social reward circuits in the VTA. Nat. Neurosci..

[141] Stefansson H., Sigurdsson E., Steinthorsdottir V., Bjornsdottir S., Sigmundsson T., Ghosh S., Brynjolfsson J., Gunnarsdottir S., Ívarsson Ó., Chou T.T. (2002). Neuregulin 1 and Susceptibility to Schizophrenia. Am. J. Hum. Genet..

[142] Yang J.Z., Si T.M., Ruan Y., Ling Y.S., Han Y.H., Wang X.L., Zhou M., Zhang H.Y., Kong Q.C., Liu C. (2003). Association study of neuregulin 1 gene with schizophrenia. Mol. Psychiatry.

[143] Thomson P.A., Christoforou A., Morris S.W., Adie E., Pickard B.S., Porteous D.J. (2007). Association of Neuregulin 1 with schizophrenia and bipolar disorder in a second cohort from the Scottish population. Mol. Psychiatry.

[144] Van Beveren N.J., Krab L.C., Swagemakers S., Buitendijk G.H., Boot E., van der Spek P., Elgersma Y., van Amelsvoort T.A. (2012). Functional gene-expression analysis shows involvement of schizophrenia-relevant pathways in patients with 22q11 deletion syndrome. PLoS ONE.

[145] Kasnauskiene J., Ciuladaite Z., Preiksaitiene E., Utkus A., Peciulyte A., Kučinskas V. (2013). A new single gene deletion on 2q34: ERBB4 is associated with intellectual disability. Am. J. Med. Genet. A.

[146] Abbasy S., Shahraki F., Haghighatfard A., Qazvini M.G., Rafiei S.T., Noshadirad E., Farhadi M., Rezvani H., Shiryazdi A.A., Ghamari R. (2018). Neuregulin1 types mRNA level changes in autism spectrum disorder, and is associated with deficit in executive functions. EBioMedicine.

[147] Esnafoglu E. (2018). Levels of peripheral Neuregulin 1 are increased in non-medicated autism spectrum disorder patients. J. Clin. Neurosci..

[148] Woo R.S., Lee J.H., Yu H.N., Song D.Y., Baik T.K. (2010). Expression of ErbB4 in the apoptotic neurons of Alzheimer’s disease brain. Anat. Cell. Biol..

[149] Chaudhury A.R., Gerecke K.M., Wyss J.M., Morgan D.G., Gordon M.N., Carroll S.L. (2003). Neuregulin-1 and erbB4 immunoreactivity is associated with neuritic plaques in Alzheimer disease brain and in a transgenic model of Alzheimer disease. J. Neuropathol. Exp. Neurol..

[150] Go R.C.P., Perry R.T., Wiener H., Bassett S.S., Blacker D., Devlin B., Sweet R.A. (2005). Neuregulin-1 polymorphism in late onset Alzheimer’s disease families with psychoses. Am. J. Med Genet. Part B Neuropsychiatr. Genet..

[151] Wang K.S., Xu N., Wang L., Aragon L., Ciubuc R., Arana T.B., Mao C., Petty L., Briones D., Branda B.S. (2014). NRG3 gene is associated with the risk and age at onset of Alzheimer disease. J. Neural Transm. (Vienna).

[152] Bertram I., Bernstein H.G., Lendeckel U., Bukowska A., Dobrowolny H., Keilhoff G., Kanakis D., Mawrin C., Bielau H., Falkai P. (2007). Immunohistochemical evidence for impaired neuregulin-1 signaling in the prefrontal cortex in schizophrenia and in unipolar depression. Ann. N. Y. Acad. Sci..

[153] Milanesi E., Minelli A., Cattane N., Cattaneo A., Mora C., Barbon A., Mallei A., Popoli M., Florio V., Conca A. (2012). ErbB3 mRNA leukocyte levels as a biomarker for major depressive disorder. BMC Psychiatry.

[154] Mahar I., Labonte B., Yogendran S., Isingrini E., Perret L., Davoli M.A., Rachalski A., Giros B., Turecki G., Mechawar N. (2017). Disrupted hippocampal neuregulin-1/ErbB3 signaling and dentate gyrus granule cell alterations in suicide. Transl. Psychiatry.

[155] Depboylu C., Höllerhage M., Schnurrbusch S., Brundin P., Oertel W.H., Schrattenholz A., Höglinger G.U. (2012). Neuregulin-1 receptor tyrosine kinase ErbB4 is upregulated in midbrain dopaminergic neurons in Parkinson disease. Neurosci. Lett..

[156] Hama Y., Yabe I., Wakabayashi K., Kano T., Hirotani M., Iwakura Y., Utsumi J., Sasaki H. (2015). Level of plasma neuregulin-1 SMDF is reduced in patients with idiopathic Parkinson’s disease. Neurosci. Lett..

[157] Fisher M.L., Loukola A., Kaprio J., Turner J.R. (2015). Role of the Neuregulin Signaling Pathway in Nicotine Dependence and Co-morbid Disorders. Int. Rev. Neurobiol..

[158] Gupta R., Qaiser B., He L., Hiekkalinna T.S., Zheutlin A.B., Therman S., Ollikainen M., Ripatti S., Perola M., Salomaa V. (2017). Neuregulin signaling pathway in smoking behavior. Transl. Psychiatry.

[159] Vaht M., Laas K., Kiive E., Parik J., Veidebaum T., Harro J. (2017). A functional neuregulin-1 gene variant and stressful life events: Effect on drug use in a longitudinal population-representative cohort study. J. Psychopharmacol..

